# An Improved Cockroach Swarm Optimization

**DOI:** 10.1155/2014/375358

**Published:** 2014-05-14

**Authors:** I. C. Obagbuwa, A. O. Adewumi

**Affiliations:** School of Mathematics, Statistics and Computer Science, University of KwaZulu-Natal, Westville, Durban 4000, South Africa

## Abstract

Hunger component is introduced to the existing cockroach swarm optimization (CSO) algorithm to improve its searching ability and population diversity. The original CSO was modelled with three components: chase-swarming, dispersion, and ruthless; additional hunger component which is modelled using partial differential equation (PDE) method is included in this paper. An improved cockroach swarm optimization (ICSO) is proposed in this paper. The performance of the proposed algorithm is tested on well known benchmarks and compared with the existing CSO, modified cockroach swarm optimization (MCSO), roach infestation optimization RIO, and hungry roach infestation optimization (HRIO). The comparison results show clearly that the proposed algorithm outperforms the existing algorithms.

## 1. Introduction

Swarm intelligence (SI) is a method of computing whereby simple decentralized agents get information by interacting locally with one another and their environment [1]. The local information received is not controlled centrally; local interaction of agents results in amazing and emergent global patterns which can be adopted for solving problems [1].

SI algorithms draw inspiration from insects and animals social behaviour and have been proven in literature to be efficient in solving global optimization problems. Examples of existing SI algorithms include particle swarm optimization (PSO), ant colony optimization (ACO), and bee colony optimization (BCO). PSO based on bird social behaviour, introduced by Kennedy and Eberhart [2], has been applied to several problems, including power and management processes [3, 4] and combinatorial optimization problem in [5]. ACO based on ant social behaviour, introduced by Dorigo [6], has been applied to problems such as vehicle routing problem [7] and network routing problem [8]. BCO based on bees social behaviour, introduced by Pham et al. [9], has been applied to real world problems by Karaboga and his research group [10–12].

One of the recent developments in SI is cockroach optimization [13–16]. Cockroach belongs to Insecta Blattodea, abodes in warm, dark, and moist shelters, and exhibits habits which include chasing, swarming, dispersing, being ruthless and omnivorous, and food searching. Cockroaches interact with peers and respond to their immediate environment and make decisions based on their interaction such as selecting shelter, searching for food sources and friends, dispersing when danger is noticed, and eating one another when food is scarce.

The original cockroach swarm optimization (CSO) algorithm, introduced by Zhaohui and Haiyan [14], was modified by ZhaoHui with the introduction of inertial weight [15]. CSO algorithms [14, 15] mimic chase swarming, dispersion, and ruthless social behaviour of cockroaches.

Global optimization problems are considered as very hard problems, ever increasing in complexity. It became necessary to design better optimization algorithms; this necessitated the design of a better cockroach algorithm. This paper extends MCSO with the introduction of another social behaviour called hunger behaviour. Hunger behaviour prevents local optimum and enhances diversity of population. An improved cockroach swarm optimization (ICSO) is presented in this paper.

The organization of this paper is as follows: Section 2 presents CSO, MCSO, and ICSO models with algorithmic steps; Section 3 shows the experiments carried out and results obtained; the paper is summarised in Section 4.

## 2. Cockroach Swarm Optimization

CSO algorithm is a population based global optimization algorithm which has been applied to problems in literature including [17–19]. CSO [14] models are given as follows.

*(1) Chase-Swarming Behaviour*.

(1) $$\begin{array}{c} x_{i}=\{\begin{array}{cc} x_{i}+\text{step}\cdot \text{rand}\cdot \left(p_{i}-x_{i}\right), & x_{i}\neq p_{i}\\ x_{i}+\text{step}\cdot \text{rand}\cdot \left(p_{g}-x_{i}\right), & x_{i}=p_{i}, \end{array} \end{array}$$

where *x*_*i*_ is the cockroach position, step is a fixed value, rand is a random number within [0,1], *p*_*i*_ is the personal best position, and *p*_*g*_ is the global best position. Consider

(2) $$\begin{array}{c} p_{i}={\text{Opt}}_{j}\left\{x_{j},\left|x_{i}-x_{j}\right|\leq \text{visual}\right\}, \end{array}$$

where perception distance visual is a constant, *j* = 1,2,…, *N*, *i* = 1,2,…, *N*. Consider

(3) $$\begin{array}{c} p_{g}={\text{Opt}}_{i}\left\{x_{i}\right\} \end{array}$$

*(2) Dispersion Behaviour.*

(4) $$\begin{array}{c} x_{i}=x_{i}+\text{rand}\left(1,D\right),\;i=1,2,\ldots ,N, \end{array}$$

where rand(1, *D*) is a *D*-dimensional random vector that can be set within a certain range.

*(3) Ruthless Behaviour.*

(5) $$\begin{array}{c} x_{k}=p_{g}, \end{array}$$

where *k* is a random integer within [1, *N*] and *p*_*g*_ is the global best position.

### 2.1. Modified Cockroach Swarm Optimization

ZhaoHui presented a modified cockroach swarm optimization (MCSO) [15] with the introduction of inertial weight to chase swarming component of original CSO as shown below. Other models remain as in original CSO.

Chase-swarming behaviour is as follows:

(6) $$\begin{array}{c} x_{i}=\{\begin{array}{cc} w\cdot x_{i}+\text{step}\cdot \text{rand}\cdot \left(p_{i}-x_{i}\right), & x_{i}\neq p_{i}\\ w\cdot x_{i}+\text{step}\cdot \text{rand}\cdot \left(p_{g}-x_{i}\right), & x_{i}=p_{i}, \end{array} \end{array}$$

where *w* is an inertial weight which is a constant.

### 2.2. Improved Cockroach Swarm Optimization

In this paper, MCSO is extended with additional component called hunger behaviour.

#### 2.2.1. Hunger Behaviour

At interval of time, when cockroach is hungry, it migrates from its comfortable shelter and friends company to look for food [13, 20]. Hunger behaviour is modelled using partial differential equation (PDE) migration techniques [21]. Cockroach migrates from its shelter to any available food source *x*_food_ within the search space. A threshold hunger is defined, when cockroach is hungry and threshold hunger is reached; it migrates to food source. Hunger behaviour prevents local optimum and enhances diversity of population.

PDE migration equation is described by Kerckhove [21]:

(7) $$\begin{array}{c} \frac{\partial u}{\partial t}=-c\frac{\partial u}{\partial x} \end{array}$$

with *u*(0, *x*) = *u*_0_(*x*).

Parameter *c* is the controlling speed of the migration. *u* is the population size, *t* is time, and *x* is location or position. *u*(*t*, *x*) is the population size at time *t* in location *x* with *u*(0, *x*) = *u*_0_(*x*) being the initial population distribution. Consider

(8) ∂u∂t=−c∂u∂x,∂u∂t+c∂u∂x=0.

The characteristic equations are

(9) dt1=dxc=du0,dx−cdt=0.

By integration, we have

(10) x−ct=α,u=u(α),u=u(x−ct),u[t,x]=u0[−ct+x].

Consider displacement = speed × time.

In *u*_0_(*x* − *ct*), *u*_0_(*x*) displaces *ct*.

*u*_0_(*x* − *ct*) satisfies migration equation at any initial population distribution *u*_0_(*x*) [21].

Hunger behaviour is modelled as follows:

If (hunger = = *t*_hunger_)

(11) $$\begin{array}{c} x_{i}=x_{i}+\left(x_{i}-ct\right)+x_{\text{food}}, \end{array}$$

where *x*_*i*_ denotes cockroach position, (*x*_*i*_ − *ct*) denotes cockroach migration from its present position, *c* is a constant which controls migration speed at time *t*, *x*_food_ denotes food location, *t*_hunger_ denotes hunger threshold, and hunger is a random number [0,1].

#### 2.2.2. Improved Cockroach Swarm Optimization Models

*(1) Chase-Swarming Behaviour*.

(12) $$\begin{array}{c} x_{i}=\{\begin{array}{cc} w\cdot x_{i}+\text{step}\cdot \text{rand}\cdot \left(p_{i}-x_{i}\right), & x_{i}\neq p_{i},\\ w\cdot x_{i}+\text{step}\cdot \text{rand}\cdot \left(p_{g}-x_{i}\right), & x_{i}=p_{i}, \end{array} \end{array}$$

where *w* is an inertial weight which is a constant, step is a fixed value, rand is a random number within [0,1], *p*_*i*_ is the personal best position, and *p*_*g*_ is the global best position. Consider

(13) $$\begin{array}{c} p_{i}={\text{Opt}}_{j}\left\{x_{j},\left|x_{i}-x_{j}\right|\leq \text{visual}\right\}, \end{array}$$

where perception distance visual is a constant, *j* = 1,2,…, *N*, *i* = 1,2,…, *N*. Consider

(14) $$\begin{array}{c} p_{g}={\text{Opt}}_{i}\left\{x_{i}\right\} \end{array}$$

*(2) Hunger Behaviour*. If hunger = = *t*_hunger_,

(15) $$\begin{array}{c} x_{i}=x_{i}+\left(x_{i}-ct\right)+x_{\text{food}}, \end{array}$$

where *x*_*i*_ denotes cockroach position, (*x*_*i*_ − *ct*) denotes cockroach migration from its present position, *c* is a constant which controls migration speed at time *t*, *x*_food_ denotes food location, *t*_hunger_ denotes hunger threshold, and hunger is a random number within [0,1].

*(3) Dispersion Behaviour*.

(16) $$\begin{array}{c} x_{i}=x_{i}+\text{rand}\left(1,D\right),\;i=1,2,\ldots ,N, \end{array}$$

where rand(1, *D*) is a *D*-dimensional random vector that can be set within a certain range.

*(4) Ruthless Behaviour*.

(17) $$\begin{array}{c} x_{k}=p_{g}, \end{array}$$

where *k* is a random integer within [1, *N*] and *p*_*g*_ is the global best position.

The algorithm for ICSO is illustrated in Algorithm 1 and its computational steps given as follows.

1. Initialise cockroach swarm with uniform distributed random numbers and set all parameters with values.
2. Find *p*_*i*_ and *p*_*g*_ using (12) and (13).
3. Perform chase-swarming using (11).
4. Perform hunger behaviour using  (14)
5. Perform dispersion behaviour using  (15).
6. Perform ruthless behaviour using  (16).
7. Repeat the loop until stopping criterion is reached.

Series of experiments are conducted in Section 3 using established global optimization problems to test ICSO performance. The performance of ICSO is compared with that of existing algorithms RIO, HRIO, CSO, and MCSO.

## 3. Simulation Studies

The speed, accuracy, robustness, stability, and searching capabilities of ICSO are evaluated in this section with 23 benchmark test functions. The test functions were adopted from [22–24]; any further information about the test functions can be found in these references. The test functions are of different characteristics such as unimodal (*U*), multimodal (*M*), separable (*S*), and nonseparable (*N*).  Table 1 of this paper shows the test functions used, whose problem ranges from 2 to 30 in dimension as in [22–24].

All algorithms were implemented in MATLAB 7.14 (R2012a) and run on a computer with 2.30 GHz processor with 4.00 GB of RAM. Experimental setting of [13–15] is used for the experiments of this paper; experiment runs 20 times with maximum iteration 1000, perception distance visual = 5, the largest step was step = 2, and inertia weight was *w* = 0.618; we defined hunger threshold *t*_hunger_ = 0.5 and hunger as a randomly generated number [0,1] in each iteration for ICSO. Cockroach parameters [13] are used for RIO and HRIO; *c*_0_ = 0.7 and *c*_max⁡_ = 1.43, hunger threshold *t*_hunger_ = 100, and hunger as randomly generated number [0, (*t*_hunger_ − 1)]. Cockroach population size *N* = 50 is used in this paper for all the algorithms. Further details about RIO, HRIO, CSO, and MSCO can be found in [13–15].

ICSO along with similar algorithms, that is, CSO, MSCO, RIO, and HRIO, was implemented with several simulation experiments conducted and reported. Success rate, average and best fitness, standard deviation (STD), and execution time in seconds are used as performance measure for comparative purpose (see Tables 2, 3, and 4 of this paper).

ICSO locates minimum values for the tested benchmark problems such as Bohachevsky, Rastrigin, Easom, Schaffer, Step, and Storn's Tchebychev problems as shown in Tables 2, 3, and 4. The comparison of the average performance of ICSO with that of RIO, HRIO, CSO, and MCSO is shown in Table 5; the comparison result clearly shows that ICSO outperforms other algorithms. Similarly, the best performance of ICSO with that of RIO, HRIO, CSO, and MCSO is shown in Table 6; ICSO has better performance than others.

ICSO algorithm has consistent performance in each iteration. This is proved by very low standard deviation of the average optimal recoded during experiments. The ICSO average optimal STD is compared with the STD of RIO, HRIO, CSO, and MCSO in Table 7. ICSO has better minimum STD than others.

ICSO locates good solutions in each experiment; this is proved by the success rate of the algorithm.  Table 8 shows the comparison of the success rate of the proposed algorithm with the existing algorithms RIO, HRIO, CSO, and MCSO. ICSO has 100% success rate in all test functions except Rosenbrock.

ICSO utilizes minimum time in executing the selected test function.  Table 9 shows the comparison of the execution time of ICSO and that of RIO, HRIO, CSO, and MCSO; ICSO is shown to have utilized minimum time.

To determine the significant difference between the performance of the proposed algorithm and the existing algorithms, test statistic of Jonckheere-Terpstra (J-T) test was conducted using the statistical package for the social science (SPSS). The Null hypothesis test for J-T test is that there is no difference among several independent groups. As the usual practice in most literature, *P* value threshold value for hypothesis test was set to 0.05. If *P* value is less than 0.05, the Null is rejected which means there is significant difference between the groups. Otherwise the Null hypothesis is accepted.  Table 10 shows the result of J-T test; *P* value (Asymp. Sig.) was computed to be 0.001. The *P* value is less than the threshold value 0.05; therefore, there is significant difference in performance of ICSO and that of RIO, HRIO, CSO, and MCSO for benchmarks evaluated.

Effect size of the significant difference is the measure of the magnitude of the observed effect. The effect size *r*, (1 > *r* < 0) of the significant difference of J-T test, was calculated as

(18) $$\begin{array}{c} r=\frac{Z}{\sqrt{N}}, \end{array}$$

where *Z* is the standard data of J-T statistic as shown in Table 10, *N* is the total number of samples, and *N* = 114. Consider

(19) $$\begin{array}{c} Z=\frac{x-\mu }{\sigma }, \end{array}$$

where *x* denotes observed J-T statistic, *μ* denotes the mean J-T statistic, and *σ* denoted the standard deviation of J-T statistic. Consider

(20) Z=1952−2599199.355=−3.245,r=−3.245114=−0.3.

The distance between the observed data and the mean in units of standard deviation is absolute value of |*Z*| (*Z* is negative when observed data is below the mean and positive when above). The effect size 0.3 is of medium size, using Cohen's guideline on effect size [25, 26]. The statistics of 0.3 effect size shows that there is significant difference of medium magnitude between proposed algorithm and existing algorithms.

## 4. Conclusion

Cockroach swarm optimization algorithm is extended in this paper with a new component called hunger component. Hunger component enhances the algorithm diversity and searching capability. An improved cockroach swarm optimization algorithm is proposed. The efficiency of the proposed algorithm is shown through empirical studies where its performance was compared with that of existing algorithms, that is, CSO, MSCO, RIO, and HRIO. Results show its outstanding performance compared to the existing algorithms. Application of the algorithm to real life problems can be considered in further studies.

## Figures and Tables

**Algorithm 1:**
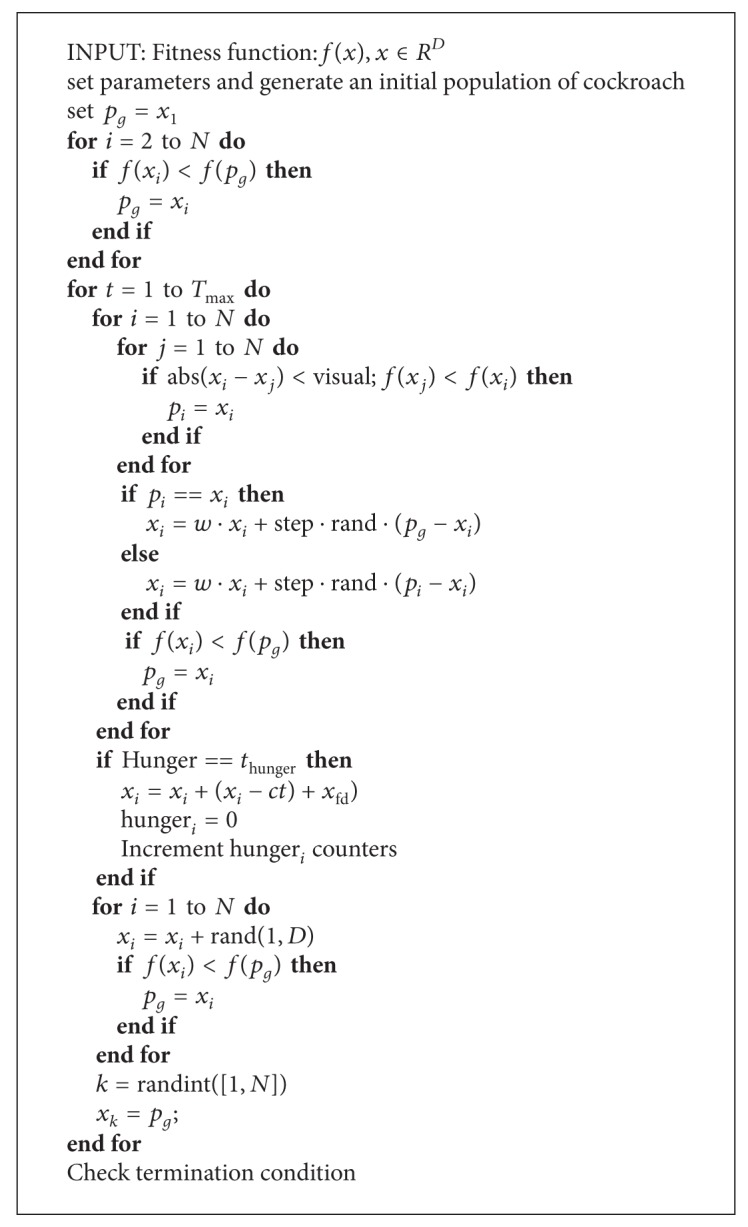
An improved cockroach swarm optimization algorithm.

**Table 1:** Benchmark test functions.

Number	Range	*D*	C	Functions	Description
1	[−100, 100]	30	US	Step	$f(x)=\overset{n}{\underset{i=1}{\sum }}{\left(\left\lfloor x_{i}+0.5\right\rfloor \right)}^{2}$
2	[−100, 100]	30	US	Sphere	$f(x)=\overset{n}{\underset{i=1}{\sum }}x_{i}^{2}$
3	[−10, 10]	30	US	Sumsquares	$f(x)=\overset{n}{\underset{i=1}{\sum }}ix_{i}^{2}$
4	[−100, 100]	2	MS	Bohachevsky1	*f*(*x*) = *x*_1_^2^ + 2*x*_2_^2^ − 0.3cos⁡(3π*x*_1_) − 0.4cos⁡(4π*x*_2_) + 0.7
5	[−100, 100]	2	MN	Bohachevsky2	*f*(*x*) = *x*_1_^2^ + 2*x*_2_^2^ − 0.3cos⁡(3π*x*_1_)(4π*x*_2_) + 0.3
6	[−100, 100]	2	MN	Bohachevsky3	*f*(*x*) = *x*_1_^2^ + 2*x*_2_^2^ − 0.3cos⁡(3π*x*_1_ + 4π*x*_2_) + 0.3
7	[0, 180]	20	UN	Sinusoidal20	$f(x)=-\left[A\prod_{i=1}^{n}\sin\left(x_{i}-z\right)+\prod_{i=1}^{n}\sin\left(B\left(x_{i}-z\right)\right)\right]$
					*A* = 2.5, *B* = 5, *z* = 30
8	[−100, 100]	30	UN	Quadric	$f(x)=\overset{n}{\underset{i=1}{\sum }}{\left(\overset{n}{\underset{i=1}{\sum }}x_{j}\right)}^{2}$
9	[−100, 100]	2	UN	Easom	*f*(*x*) = −cos⁡*x*_1_cos⁡*x*_2_ · exp⁡(−(*x*_1_−π)^2^)exp⁡(−(*x*_2_ − π)^2^)
10	[−10, 10]	2	UN	Matyas	*f*(*x*) = 0.26(*x*_1_ + *x*_2_) − 0.48*x*_1_ *x*_2_
11	[−5, 10]	10	UN	Zakharov	$f(x)=\overset{n}{\underset{i=1}{\sum }}{\left(x_{i}\right)}^{2}+{\left(\overset{n}{\underset{i=1}{\sum }}0.5ix_{i}\right)}^{2}+{\left(\overset{n}{\underset{i=1}{\sum }}0.5ix_{i}\right)}^{4}$
12	[−10, 10]	24	UN	Powell	$f(x)=\overset{n/k}{\underset{i=1}{\sum }}{\left(x_{4i-3}+10x_{4i-2}\right)}^{2}+5{\left(x_{4i-1}-x_{4i}\right)}^{2}+{\left(x_{4i-2}-x_{4i-1}\right)}^{4}+10{\left(x_{4i-3}-x_{4i}\right)}^{4}$
13	[−10, 10]	30	UN	Schwefel2.22	$f(x)=\overset{n}{\underset{i=1}{\sum }}\left|x_{i}\right|+\prod_{i=1}^{n}\left|x_{i}\right|$
14	[−30, 30]	30	UN	Rosenbrock	$f\left(x\right)=\overset{n-1}{\underset{i=1}{\sum }}\left[100{\left(x_{i+1}-x_{i}^{2}\right)}^{2}+{\left(x_{i}-1\right)}^{2}\right]$
15	[−5.12, 5.12]	30	MS	Rastrigin	$f(x)=\overset{n}{\underset{i=1}{\sum }}x_{i}^{2}-10\cos\left(2\pi x_{i}\right)+10$
16	[−100, 100]	2	MN	Schaffer1	$f(x)=0.5+\frac{\sin^{2}{\sqrt{x_{1}^{2}+x_{2}^{2}}}^{2}-0.5}{{\left[1+0.001\left(x_{1}^{2}+x_{2}^{2}\right)\right]}^{2}}$
17	[−100, 100]	30	MN	Schaffer2	*f*(*x*) = (*x*_1_^2^+*x*_2_^2^)^0.25^(sin⁡^2^⁡(50(*x*_1_^2^ + *x*_2_^2^)^0.1^) + 1)
18	[−600, 600]	30	MN	Griewangk	$f(x)=\frac{1}{4000}\overset{n}{\underset{i=1}{\sum }}x_{i}^{2}-\prod_{i=1}^{n}\cos\left(\frac{x_{i}}{\sqrt{i}}\right)+1$
19	[−32, 32]	30	MN	Ackley	$f(x)=-20\exp^{\left(-0.2\sqrt{\sum_{i=1}^{n}‍(x_{i}^{2}/n)}\right)}-\exp^{\left(\sum_{i=1}^{n}‍\cos\left(2\pi x_{i}/n\right)\right)}+20+e$
20	[−5, 5]	2	MN	Three hump camel back	$f(x)=2x_{1}^{2}-1.05x_{1}^{4}+\frac{1}{6}x_{1}^{6}+x_{1}x_{2}+x_{2}^{2}$
21	[−5, 5]	2	MN	Six hump camel back	$f(x)=4x_{1}^{2}-2.1x_{1}^{4}+\frac{1}{3}x_{1}^{6}+x_{1}x_{2}-4x_{2}^{2}+4x_{2}^{4}$
22	[−128, 128]^*n*^	9	UN	Storn's Tchebychev	*f*(*x*) = *p*_1_ + *p*_2_ + *p*_3_,
23	[−32768, 32768]^*n*^	17		Storn's Tchebychev	wherep1={(u-d)2if u<d0if u≥du=∑i=1n(1.2)n-ixip2={(v-d)2if v<d0if v≥dv=∑i=1n(-1.2)n-ixip3=∑j=0m{(wj-1)2if wj>1(wj+1)2if wj<-10if -1≤wj≤1wj=∑i=1n(2jm-1)n-ixi, for *n* = 9: *d* = 72.661, and *m* = 60 for *n* = 17: *d* = 10558.145, and *m* = 100.

*D*: dimension; C: characteristic; U: unimodal; S: seperable; N: non-separable.

**Table 2:** Simulation results of RIO, HRIO, CSO, MCSO, and ICSO.

SN	Fn.	Dim.	Opt.		RIO	HRIO	CSO	MCSO	ICSO
1	Boha1	2	0	Ave.	3.4405*E* − 05	3.2877*E* − 04	2.9893*E*02	3.5153*E* − 09	0.0000
				STD	2.5963*E* − 05	3.0334*E* − 04	5.0332*E*02	1.4392*E* − 08	0.0000
				Best	1.3520*E* − 07	5.2651*E* − 06	2.0651*E* − 05	0.0000	0.0000
				Success	20/20	20/20	5/20	20/20	20/20
				Time	1.137525	0.886356	23.913237	0.075212	0.097187

2	Boha2	2	0	Ave.	4.2829*E* − 05	4.6703*E* − 04	9.0941*E*02	8.4459*E* − 12	0.0000
				STD	3.0070*E* − 05	3.4047*E* − 04	1.7794*E*03	2.9240*E* − 11	0.0000
				Best	2.2910*E* − 06	9.374*E* − 06	1.3775*E* − 05	0.0000	0.0000
				Success	20/20	20/20	4/20	20/20	20/20
				Time	0.998178	0.946887	26.492095	0.072021	0.074106

3	Boha3	2	0	Ave.	5.3479*E* − 05	4.7575*E* − 04	7.4284*E*02	2.1388*E* − 14	0.0000
				STD	2.9141*E* − 05	2.3273*E* − 04	1.6739*E*03	4.8670*E* − 14	0.0000
				Best	3.1200*E* − 06	4.6981*E* − 05	2.3093*E* − 07	0.0000	0.0000
				Success	20/20	20/20	3/20	20/20	20/20
				Time	1.089920	0.885252	25.028054	0.080908	0.068189

4	3camel	2	0	Ave.	1.4962*E* − 02	4.3021*E* − 04	5.003*E*09	7.098*E* − 11	5.9853*E* − 31
				STD	6.6769*E* − 02	2.8371*E* − 04	1.7137*E*10	3.0201*E* − 10	2.5457*E* − 30
				Best	1.1739*E* − 06	2.2449*E* − 05	1.7642*E* − 05	3.1395*E* − 19	2.2320*E* − 53
				Success	19/20	20/20	12/20	20/20	20/20
				Time	4.231533	0.794983	18.281683	0.104132	0.078845

5	6camel	2	−1.03163	Ave.	−4.3522*E* − 01	−4.7652*E* − 01	1.5763*E*05	−1.0263*E* − 08	−2.9798*E* − 25
				STD	3.3322*E* − 01	3.1284*E* − 01	7.0503*E*05	4.4391*E* − 08	1.3325*E* − 24
				Best	−1.0215	−1.0034	−9.4052*E* − 01	−1.9879*E* − 07	−5.9589*E* − 24
				Success	20/20	20/20	19/20	20/20	20/20
				Time	0.406355	0.330198	5.723039	0.0945856	0.086637

6	Easom	2	−1	Ave.	−1	−1	−4.3165*E* − 01	−1	−1
				STD	3.7518*E* − 02	2.1031*E* − 02	3.4470*E* − 01	1.4897*E* − 08	4.4116*E* − 17
				Best	−1	−1	−1	−1	−1
				Success	20/20	20/20	20/20	20/20	20/20
				Time	0.124022	0.107303	0.106738	0.077179	0.092393

7	Matyax	2	0	Ave.	4.9470*E* − 05	3.2297*E* − 04	7.5712	2.6876*E* − 13	4.0732*E* − 35
				STD	3.0244*E* − 05	2.6018*E* − 04	1.1247*E*01	8.9347*E* − 13	1.8125*E* − 34
				Best	6.2897*E* − 06	1.2684*E* − 05	8.8777*E* − 06	6.6695*E* − 21	1.1292*E* − 55
				Success	20/20	20/20	11/20	20/20	20/20
				Time	0.973322	0.711734	13.559576	0.88536	0.076693

8	Schaffer1	2	−1	Ave.	−1.9069	−1.6211	−2.9174*E* − 01	−1	−1
				STD	7.0381*E* − 01	5.9214*E* − 01	7.5142*E* − 01	5.9575*E* − 07	4.1325*E* − 15
				Best	−2.7458	−2.7164	−2.7438	−1	−1
				Success	20/20	20/20	20/20	20/20	20/20
				Time	0.109048	0.086433	0.119076	0.072400	0.081599

9	Schaffer2	2	0	Ave.	2.0179*E* − 03	1.6566*E* − 03	7.1618	3.3168*E* − 04	2.2149*E* − 09
				STD	2.6407*E* − 03	1.4451*E* − 03	5.3095	3.0328*E* − 04	2.9483*E* − 09
				Best	6.2423*E* − 05	4.1422*E* − 04	2.8354*E* − 01	1.5810*E* − 05	1.9383*E* − 14
				Success	2/20	13/20	0/20	20/20	20/20
				Time	62.567654	31.415836	29.194283	0.084127	0.082320

Dim. denotes dimension. Opt. denotes optimum value. Boha1 denotes Bohachevsky1. Boha2 denotes Bohachevsky2. Boha3 denotes Bohachevsky3. 3camel denotes three hump camel back. 6camel denotes six hump camel back.

**Table 3:** Simulation results of RIO, HRIO, CSO, MCSO, and ICSO.

SN	Fn.	Dim.	Opt.		RIO	HRIO	CSO	MCSO	ICSO
10	Sphere	30	0	Ave.	2.2168*E* − 05	1.6676*E* − 04	1.8123*E*02	1.5201*E* − 12	3.3448*E* − 34
				STD	2.4528*E* − 05	2.4018*E* − 04	8.1048*E*02	6.7224*E* − 12	1.3324*E* − 33
				Best	5.7627*E* − 09	5.5635*E* − 08	4.9195*E* − 07	2.9978*E* − 24	2.8205*E* − 54
				Success	20/20	20/20	19/20	20/20	20/20
				Time	0.617544	0.557871	25.378161	0.82512	0.199373

11	Rastrigin	30	0	Ave.	3.8135*E* − 05	3.2150*E* − 04	3.6022*E*03	9.1994*E* − 11	0.0000
				STD	3.4436*E* − 05	3.0003*E* − 04	5.5728*E*03	3.9456*E* − 10	0.0000
				Best	2.7098*E* − 07	2.1450*E* − 07	3.1340*E* − 04	0.0000	0.0000
				Success	20/20	20/20	5/20	20/20	20/20
				Time	0.956329	0.826770	71.811170	0.175563	0.369987

12	Rosenbrock	30	0	Ave.	2.5281*E*06	3.3571*E*06	9.5067*E*11	2.9000*E*01	2.9000*E*01
				STD	4.0528*E*06	7.1150*E*06	2.2713*E*12	0.0000	0.0000
				Best	1.6773*E*04	3.7562*E*04	4.4068*E*01	2.9000*E*01	2.9000*E*01
				Success	0/20	0/20	0/20	0/20	0/20
				Time	126.618734	127.469638	81.361663	76.084929	78.572185

13	Ackley	30	0	Ave.	2.0001*E*01	2.0005*E*01	1.9222*E*01	5.1593*E* − 06	1.0651*E* − 15
				STD	3.0455*E* − 03	1.5671*E* − 02	5.8258	1.9149*E* − 05	7.9441*E* − 16
				Best	2.0001*E*01	1.9998*E*01	2.0133*E*01	6.4623*E* − 09	8.1818*E* − 16
				Success	0/20	0/20	0/20	20/20	20/20
				Time	122.216187	117.635854	82.227210	0.235012	0.192339

14	Quadric	30	0	Ave.	2.4498*E* − 05	2.2711*E* − 04	3.4991*E* − 04	4.4754*E* − 13	7.2183*E* − 28
				STD	2.7957*E* − 05	2.3635*E* − 04	3.3725*E* − 04	1.9751*E* − 12	3.2218*E* − 27
				Best	1.1360*E* − 08	5.8230*E* − 07	4.1551*E* − 08	5.6309*E* − 23	5.910*E* − 52
				Success	20/20	20/20	20/20	20/20	20/20
				Time	0.718785	0.512242	31.075809	0.247456	0.227244

15	Schwefel2.22	30	0	Ave.	2.3131*E*02	2.4395*E*02	2.9013*E*54	6.3587*E* − 06	6.0407*E* − 16
				STD	1.3193*E*02	1.2341*E*02	1.2971*E*55	1.1936*E* − 05	1.2203*E* − 15
				Best	6.7400*E*01	1.7354*E*01	3.6854*E*01	5.9410*E* − 08	5.1670*E* − 24
				Success	0/20	0/20	0/20	20/20	20/20
				Time	128.445013	127.084387	79.924516	0.217104	0.219296

16	Griewangk	30	0	Ave.	7.9510*E* − 01	7.7746*E* − 01	2.6148*E*01	3.3151*E* − 11	0.0000
				STD	3.7583*E* − 01	2.5454*E* − 01	3.6626*E*01	1.4672*E* − 10	0.0000
				Best	2.9324*E* − 01	3.2031*E* − 01	6.3912*E* − 05	0.0000	0.0000
				Success	0/20	0/20	5/20	20/20	20/20
				Time	126.872461	126.210153	70.852376	0.211351	0.210934

17	Sumsquare	30	0	Ave.	1.9818*E*03	4.6771*E*03	9.0499*E*05	4.2446*E* − 11	1.5600*E* − 24
				STD	2.8370*E*03	6.7104*E*03	1.0253*E*06	1.2930*E* − 10	6.9785*E* − 24
				Best	1.6463*E*01	2.0516*E*02	1.8730*E*02	1.49990*E* − 16	1.3765*E* − 47
				Success	0/20	0/20	0/20	20/20	20/20
				Time	122.748646	125.154349	78.809270	0.273780	0.236129

18	Sinusoidal	30	−3.5	Ave.	−4.2587*E* − 01	−3.7898*E* − 01	−2.449	−3.1030	−3.1030
				STD	2.6632*E* − 01	1.9791*E* − 01	1.0203	5.0473*E* − 05	1.9436*E* − 14
				Best	−1.1922	−8.3111*E* − 01	−3.3087	−3.1032	−3.1030
				Success	20/20	20/20	20/20	20/20	20/20
				Time	0.204559	0.240200	0.234205	0.200361	0.217635

Dim. denotes dimension. Opt. denotes optimum value.

**Table 4:** Simulation results of RIO, HRIO, CSO, MCSO, and ICSO.

SN	Function	Dim.	Opt.		RIO	HRIO	CSO	MCSO	ICSO
19	Zakharov	30	0	Ave.	1.0167*E*04	1.0216*E*04	6.3663*E*18	2.3878*E* − 09	4.1579*E* − 26
				STD	3.8643*E*03	5.1012*E*03	2.2732*E*19	8.8529*E* − 09	1.8549*E* − 25
				Best	2.6634*E*03	2.3151*E*03	1.3578*E*09	2.0954*E* − 15	6.3965*E* − 57
				Success	0/20	0/20	0/20	20/20	20/20
				Time	115.192226	114.691827	79.926232	0.205280	0.259202

20	Step	30	0	Ave.	0.0000	0.0000	2.0004*E*04	0.0000	0.0000
				STD	0.0000	0.0000	8.4815*E*04	0.0000	0.0000
				Best	0.0000	0.0000	0.0000	0.0000	0.0000
				Success	20/20	20/20	16/20	20/20	20/20
				Time	0.686403	0.633264	39.136696	0.239525	0.225102

21	Powell	24	0	Ave.	1.8348*E* − 03	3.7434*E* − 03	1.0840*E*08	2.6031*E* − 12	1.8207*E* − 24
				STD	1.6248*E* − 03	6.1711*E* − 03	4.1180*E*08	6.9959*E* − 12	5.6824*E* − 24
				Best	9.6693*E* − 05	6.8033*E* − 04	5.2392*E*01	1.2287*E* − 19	1.2265*E* − 54
				Success	2/20	12/20	0/20	20/20	20/20
				Time	122.796991	92.876086	74.794730	1.527170	0.853751

22	ST	9	0	Ave.	0.0000	0.0000	0.0000	0.0000	0.0000
				STD	0.0000	0.0000	0.0000	0.0000	0.0000
				Best	0.0000	0.0000	0.0000	0.0000	0.0000
				Success	20/20	20/20	20/20	20/20	20/20
				Time	0.435911	0.426320	0.437944	0.431122	0.436741

23	ST	17	0	Ave.	0.0000	0.0000	0.0000	0.0000	0.0000
				STD	0.0000	0.0000	0.0000	0.0000	0.0000
				Best	0.0000	0.0000	0.0000	0.0000	0.0000
				Success	20/20	20/20	20/20	20/20	20/20
				Time	1.066161	1.052169	1.159830	1.089657	1.147114

Dim. denotes dimension. Opt. denotes optimum value.

**Table 5:** Comparison of average performance of RIO, HRIO, CSO, MCSO, and ICSO.

SN	Function	RIO	HRIO	CSO	MCSO	ICSO	Optimum
1	Bohachevsky1	3.4405*E* − 05	3.2877*E* − 04	2.9893*E*02	3.5153*E* − 09	**0.0000**	0
2	Bohachevsky2	4.2829*E* − 05	4.6703*E* − 04	9.0941*E*02	8.4459*E* − 12	**0.0000**	0
3	Bohachevsky3	5.3479*E* − 05	4.7575*E* − 04	7.4284*E*02	2.1388*E* − 14	**0.0000**	0
4	3 Hump camel back	1.4962*E* − 02	4.3021*E* − 04	5.003*E*09	7.098*E* − 11	5.9853**E** − 31	0
5	6 Hump camel back	−4.3522*E* − 01	−4.7652*E* − 01	1.5763*E*05	−1.0263*E* − 08	−2.9798**E** − 25	−1.03163
6	Easom	−1	−1	−4.3165*E* − 01	−1	−1	−1
7	Matyax	4.9470*E* − 05	3.2297*E* − 04	7.5712	2.6876*E* − 13	4.0732**E** − 35	0
8	Schaffer1	−1.9069	−1.6211	−2.9174*E* − 01	−1	−1	−1
9	Schaffer2	2.0179*E* − 03	1.6566*E* − 03	7.1618	3.3168*E* − 04	2.2149**E** − 09	0
10	Sphere	2.2168*E* − 05	1.6676*E* − 04	1.8123*E*02	1.5201*E* − 12	3.3448**E** − 34	0
11	Rastrigin	3.8135*E* − 05	3.2150*E* − 04	3.6022*E*03	9.1994*E* − 11	**0.0000**	0
12	Rosenbrock	2.5281*E*06	3.3571*E*06	9.5067*E*11	2.9000**E**01	2.9000**E**01	0
13	Ackley	2.0001*E*01	2.0005*E*01	1.9222*E*01	5.1593*E* − 06	1.0651**E** − 15	0
14	Quadric	2.4498*E* − 05	2.2711*E* − 04	3.4991*E* − 04	4.4754*E* − 13	7.2183**E** − 28	0
15	Schwefel2.22	2.3131*E*02	2.4395*E*02	2.9013*E*54	6.3587*E* − 06	6.0407**E** − 16	0
16	Griewangk	7.9510*E* − 01	7.7746*E* − 01	2.6148*E*01	3.3151*E* − 11	**0.0000**	0
17	Sumsquare	1.9818*E*03	4.6771*E*03	9.0499*E*05	4.2446*E* − 11	1.5600**E** − 24	0
18	Sinusoidal	−4.2587*E* − 01	−3.7898*E* − 01	−2.449	−3.1030	−3.1030	−3.5
19	Zakharov	1.0167*E*04	1.0216*E*04	6.3663*E*18	2.3878*E* − 09	4.1579**E** − 26	0
20	Step	**0.0000**	**0.0000**	2.0004*E*04	**0.0000**	**0.0000**	0
21	Powell	1.8348*E* − 03	3.7434*E* − 03	1.0840*E*08	2.6031*E* − 12	1.8207**E** − 24	0
22	ST9	**0.0000**	**0.0000**	**0.0000**	**0.0000**	**0.0000**	0
23	ST17	**0.0000**	**0.0000**	**0.0000**	**0.0000**	**0.0000**	0

Number of good optimums		4	4	2	7	23	

ST9 denotes Storn's Tchebychev 9. ST17 denotes Storn's Tchebychev 17.

**Table 6:** Comparison of best performance of RIO, HRIO, CSO, MCSO, and ICSO.

SN	Function	RIO	HRIO	CSO	MCSO	ICSO	Optimum
1	Bohachevsky1	1.3520*E* − 07	5.2651*E* − 06	2.0651*E* − 05	**0.0000**	**0.0000**	0
2	Bohachevsky2	2.2910*E* − 06	9.374*E* − 06	1.3775*E* − 05	**0.0000**	**0.0000**	0
3	Bohachevsky3	3.1200*E* − 06	4.6981*E* − 05	2.3093*E* − 07	**0.0000**	**0.0000**	0
4	3 hump camel back	1.1739*E* − 06	2.2449*E* − 05	1.7642*E* − 05	3.1395*E* − 19	2.2320**E** − 53	0
5	6 hump camel back	−1.0215	−1.0034	−9.4052*E* − 01	−1.9879*E* − 07	5.9589**E** − 24	−1.03163
6	Easom	−1	−1	−1	−1	−1	−1
7	Matyax	6.2897*E* − 06	1.2684*E* − 05	8.8777*E* − 06	6.6695*E* − 21	1.1292**E** − 55	0
8	Schaffer1	−2.7458	−2.7164	−2.7438	−1	−1	−1
9	Schaffer2	6.2423*E* − 05	4.1422*E* − 04	2.8354*E* − 01	1.5810*E* − 05	1.9383**E** − 14	0
10	Sphere	5.7627*E* − 09	5.5635*E* − 08	4.9195*E* − 07	2.9978*E* − 24	2.8205**E** − 54	0
12	Rosenbrock	1.6773*E*04	3.7562*E*04	4.4068*E*01	2.9000**E**01	2.9000**E**01	0
14	Quadric	1.1360*E* − 08	5.8230*E* − 07	4.1551*E* − 08	5.6309*E* − 23	5.910**E** − 52	0
15	Schwefel2.22	6.7400*E*01	1.7354*E*01	3.6854*E*01	5.9410*E* − 08	5.1670**E** − 24	0
16	Griewangk	2.9324*E* − 01	3.2031*E* − 01	6.3912*E* − 05	**0.0000**	**0.0000**	0
17	Sumsquare	1.6463*E*01	2.0516*E*02	1.8730*E*02	1.49990*E* − 16	1.3765**E** − 47	0
18	Sinusoidal	−1.1922	−8.3111*E* − 01	−3.3087	−3.1032	−3.1030	−3.5
19	Zakharov	2.6634*E*03	2.3151*E*03	1.3578*E*09	2.0954*E* − 15	6.3965**E** − 57	0
20	Step	**0.0000**	**0.0000**	**0.0000**	**0.0000**	**0.0000**	0
21	Powell	9.6693*E* − 05	6.8033*E* − 04	5.2392*E*01	1.2287*E* − 19	1.2265**E** − 54	0
22	ST9	**0.0000**	**0.0000**	**0.0000**	**0.0000**	**0.0000**	0
23	ST17	**0.0000**	**0.0000**	**0.0000**	**0.0000**	**0.0000**	0

Number of good optimums		4	4	5	11	22	

ST9 denotes Storn's Tchebychev 9. ST17 denotes Storn's Tchebychev 17.

**Table 7:** Comparison of standard deviation of mean global optimum values of RIO, HRIO, CSO, MCSO, and ICSO.

SN	Function	RIO	HRIO	CSO	MCSO	ICSO
1	Bohachevsky1	2.5963*E* − 05	3.0334*E* − 04	5.0332*E*02	1.4392*E* − 08	**0.0000**
2	Bohachevsky2	3.0070*E* − 05	3.4047*E* − 04	1.7794*E*03	2.9240*E* − 11	**0.0000**
3	Bohachevsky3	2.9141*E* − 05	2.3273*E* − 04	1.6739*E*03	4.8670*E* − 14	**0.0000**
4	3 hump camel back	6.6769*E* − 02	2.8371*E* − 04	1.7137*E*10	3.0201*E* − 10	2.5457**E** − 30
5	6 hump camel back	3.3322*E* − 01	3.1284*E* − 01	7.0503*E*05	4.4391*E* − 08	1.3325**E** − 24
6	Easom	3.7518*E* − 02	2.1031*E* − 02	3.4470*E* − 01	1.4897*E* − 08	4.4116**E** − 17
7	Matyax	3.0244*E* − 05	2.6018*E* − 04	1.1247*E*01	8.9347*E* − 13	1.8125**E** − 34
8	Schaffer1	7.0381*E* − 01	5.9214*E* − 01	7.5142*E* − 01	5.9575*E* − 07	4.1325**E** − 15
9	Schaffer12	2.6407*E* − 03	1.4451*E* − 03	5.3095	3.0328*E* − 04	2.9483**E** − 09
10	Sphere	2.4528*E* − 05	2.4018*E* − 04	8.1048*E*02	6.7224*E* − 12	1.3324**E** − 33
11	Rastrigin	3.4436*E* − 05	3.0003*E* − 04	5.5728*E*03	3.9456*E* − 10	**0.0000**
12	Rosenbrock	4.0528*E*06	7.1150*E*06	2.2713*E*12	**0.0000**	**0.0000**
13	Ackley	3.0455*E* − 03	1.5671*E* − 02	5.8258	1.9149*E* − 05	7.9441**E** − 16
14	Quadric	2.7957*E* − 05	2.3635*E* − 04	3.3725*E* − 04	1.9751*E* − 12	3.2218**E** − 27
15	Schwefel2.22	1.3193*E*02	1.2341*E*02	1.2971*E*55	1.1936*E* − 05	1.2203**E** − 15
16	Griewangk	3.7583*E* − 01	2.5454*E* − 01	3.6626*E*01	1.4672*E* − 10	**0.0000**
17	Sumsquare	2.8370*E*03	6.7104*E*03	1.0253*E*06	1.2930*E* − 10	6.9785**E** − 24
18	Sinusoidal	2.6632*E* − 01	1.9791*E* − 01	1.0203	5.0473*E* − 05	1.9436**E** − 14
19	Zakharov	3.8643*E*03	5.1012*E*03	2.2732*E*19	8.8529*E* − 09	1.8549**E** − 25
20	Step	0.0000	0.0000	8.4815*E*04	**0.0000**	**0.0000**
21	Powell	1.6248*E* − 03	6.1711*E* − 03	4.1180*E*08	6.9959*E* − 12	5.6824**E** − 24
22	ST9	**0.0000**	**0.0000**	**0.0000**	**0.0000**	**0.0000**
23	ST17	**0.0000**	**0.0000**	**0.0000**	**0.0000**	**0.0000**

Number of good STD		2	2	2	4	23

ST9 denotes Storn's Tchebychev 9. ST17 denotes Storn's Tchebychev 17.

**Table 8:** Comparison of success performance of RIO, HRIO, CSO, MCSO, and ICSO.

SN	Function	RIO	HRIO	CSO	MCSO	ICSO
1	Bohachevsky1	1	1	2.5	1	1
2	Bohachevsky2	1	1	0.2	1	1
3	Bohachevsky3	1	1	0.15	1	1
4	3 hump camel back	0.95	1	0.6	1	1
5	6 hump camel back	1	1	0.95	1	1
6	Easom	1	1	1	1	1
7	Matyax	1	1	0.55	1	1
8	Schaffer1	1	1	1	1	1
9	Schaffer2	0.1	0.65	0	1	1
10	Sphere	1	1	0.95	1	1
11	Rastrigin	1	1	0.25	1	1
12	Rosenbrock	0	0	0	0	0
13	Ackley	0	0	0	1	1
14	Quadric	1	1	1	1	1
15	Schwefel2.22	0	0	0	1	1
16	Griewangk	0	0	0.25	1	1
17	Sumsquare	0	0	0	1	1
18	Sinusoidal	1	1	1	1	1
19	Zakharov	0	0	0	1	1
20	Step	1	1	0.8	1	1
21	Powell	0.1	0.6	0	1	1
22	ST9	1	1	1	1	1
23	ST17	1	1	1	1	1

Number of 100% success rates		14	15	6	22	22

ST9 denotes Storn's Tchebychev 9. ST17 denotes Storn's Tchebychev 17.

**Table 9:** Comparison of exec1ution time of RIO, HRIO, CSO, MCSO, and ICSO.

SN	Function	RIO	HRIO	CSO	MCSO	ICSO
1	Bohachevsky1	1.137525	0.886356	23.913237	**0.075212**	0.097187
2	Bohachevsky2	0.998178	0.946887	26.492095	**0.072021**	0.074106
3	Bohachevsky3	1.089920	0.885252	25.028054	0.080908	**0.068189**
4	3 hump camel back	4.231533	0.794983	18.281683	0.104132	**0.078845**
5	6 hump camel back	0.406355	0.330198	5.723039	0.0945856	**0.086637**
6	Easom	0.124022	0.107303	0.106738	**0.077179**	0.092393
7	Matyax	0.973322	0.711734	13.559576	0.88536	**0.076693**
8	Schaffer1	0.109048	0.086433	0.119076	**0.072400**	0.081599
9	Schaffer2	62.567654	31.415836	29.194283	0.084127	**0.082320**
10	Sphere	0.617544	0.557871	25.378161	0.82512	**0.199373**
11	Rastrigin	0.956329	0.826770	71.811170	**0.175563**	0.369987
12	Rosenbrock	126.618734	127.469638	81.361663	**76.084929**	78.572185
13	Ackley	122.216187	117.635854	82.227210	0.235012	**0.192339**
14	Quadric	0.718785	0.512242	31.075809	0.247456	**0.227244**
15	Schwefel2.22	128.445013	127.084387	79.924516	**0.217104**	0.219296
16	Griewangk	126.872461	126.210153	70.852376	0.211351	**0.210934**
17	Sumsquare	122.748646	125.154349	78.809270	0.273780	**0.236129**
18	Sinusoidal	0.204559	0.240200	0.234205	**0.200361**	0.217635
19	Zakharov	115.192226	114.691827	79.926232	**0.205280**	0.259202
20	Step	0.686403	0.633264	39.136696	0.239525	**0.225102**
21	Powell	122.796991	92.876086	74.794730	1.527170	**0.853751**
22	ST9	0.435911	**0.426320**	0.437944	0.431122	0.436741
23	ST17	1.066161	**1.052169**	1.159830	1.089657	1.147114

Number of minimum execution times		—	2	—	9	12

ST9 denotes Storn's Tchebychev 9. ST17 denotes Storn's Tchebychev 17.

**Table 10:** Jonckheere-Terpstra test statistics^a^.

	Fitness
Number of levels in algorithm	5
*N*	114
Observed J-T statistic	1952.000
Mean J-T statistic	2599.500
STD of J-T statistic	199.355
Standard data of J-T statistic	−3.245
Asymp. Sig. (2-tailed)	0.001

^a^Grouping variable: algorithm.
